# Redox Processes in Neurodegenerative Disease Involving Reactive Oxygen Species

**DOI:** 10.2174/157015912804143487

**Published:** 2012-12

**Authors:** Peter Kovacic, Ratnasamy Somanathan

**Affiliations:** 1 Department of Chemistry and Biochemistry, San Diego State University, San Diego CA 92182 USA; 2 Centro de Graduados e Investigación del Instituto Tecnológico de Tijuana, Apdo postal 1166, Tijuana, B.C. Mexico

**Keywords:** Redox, neurodegenerative diseases, reactive oxygen species, oxidative stress.

## Abstract

Much attention has been devoted to neurodegenerative diseases involving redox processes. This review comprises an update involving redox processes reported in the considerable literature in recent years. The mechanism involves reactive oxygen species and oxidative stress, usually in the brain. There are many examples including Parkinson’s, Huntington’s, Alzheimer’s, prions, Down’s syndrome, ataxia, multiple sclerosis, Creutzfeldt-Jacob disease, amyotrophic lateral sclerosis, schizophrenia, and Tardive Dyskinesia. Evidence indicates a protective role for antioxidants, which may have clinical implications. A multifaceted approach to mode of action appears reasonable.

## INTRODUCTION

There has been treatment of neurotoxicity and neuro-degenerative diseases involving reactive oxygen species (ROS) and oxidative stress (OS). The present review represents an update of neurodegenerative diseases based on extensive recent literature. The redox approach comprises a unifying theme which can be applied to a large number of illnesses in this class, including Parkinson’s, Huntington’s, Alzheimer’s, prions, Down’s syndrome, ataxia, multiple sclerosis, Creutzfeldt-Jacob disease, amyotrophic lateral sclerosis, schizophrenia, and tardive dyskinesia. An earlier review addressed neurodegeneration from a similar mechanistic viewpoint based on ROS-OS [1]. Hyperoxia produces toxicity, including that of the nervous system. Too many ROS can injure tissues, which can result in human diseases of a wide variety. The mammalian brain appears to be particularly sensitive to oxidative damage, one reason being the high oxygen consumption. Rises in calcium interfere with mitochondrial function (including neural), increasing formation of superoxide which can react with nitric oxide (NO) to form the potent oxidant peroxynitrite (ONOO^־^). There is accompanying lipid peroxidation. These events include generation of various neurotoxic agents. Several neurotransmitters, including dopamine, L-DOPA, serotonin, and norepinephrine can produce ROS, evidently *via *quinone/semiquinone metabolites. Iron is found throughout the brain as complexes with various proteins (see Role of Iron below). Neural membrane lipids are replete with polyunsaturated fatty acids. Products of the oxidation, such as 4-hydroxynonenal and 4-oxononenal [2] can act as sources of ROS, and are especially cytotoxic to neurons. Brain metabolism generates an abundance of hydrogen peroxide *via *SODs (superoxide dismutases are enzymes that catalyze the dismutation of superoxide into oxygen and hydrogen peroxide) and other enzymes [1]. AO defenses are modest, such as catalase levels. Brain microglia can become activated to produce superoxide, hydrogen peroxide and cytokines. Microglia and astrocytes are major players in brain inflammation which is associated with ROS [3]. Some cytochromes leak electrons during the catalytic redox cycle, thus providing ROS [1]. Another source of brain ROS is NADPH oxidase enzymes. Hemoglobin, a neurotoxin, can release heme which is a powerful promoter of lipid peroxidation. The complex of hemoglobin with NO can also generate OS [4]. The Halliwell review presents various means for defense against neurotoxins [1]. There is also reference to earlier treatment of neurodegenerative diseases. 

**Fig. (1):**
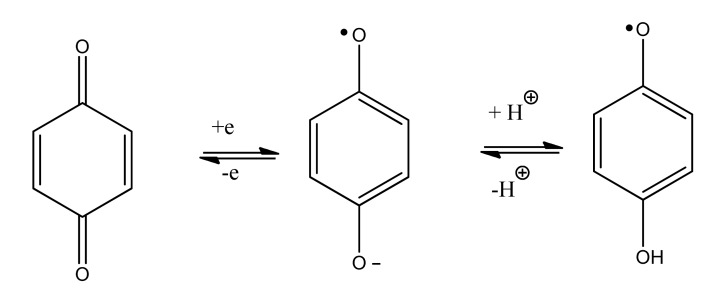
Quinone ET.

**Fig. (2):**
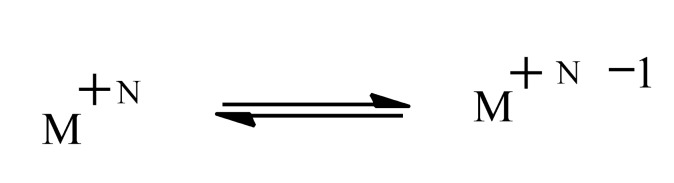
Metal ion ET.

**Fig. (3):**
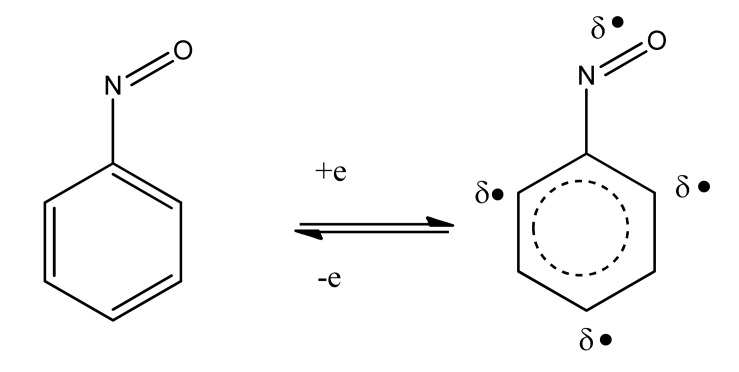
Nitrosobenzene ET.

**Fig. (4):**
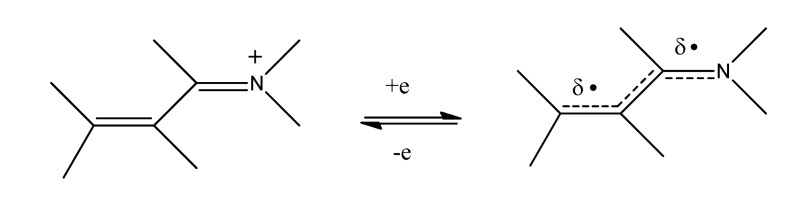
Conjugated iminium ET.

**Fig. (5):**
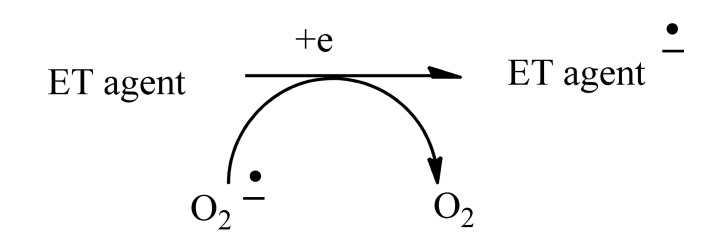
Generation of superoxide by ET.

**Fig. (6):**
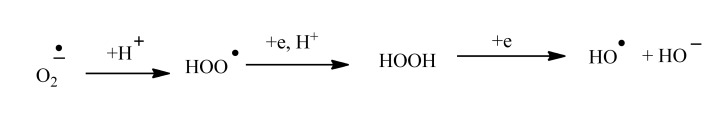
ROS from superoxide.

A broad overview of neurotoxins was presented based on electron transfer (ET), reactive oxygen species (ROS), and oxidative stress (OS) [5]. Although mode of action is complex, these aspects evidently play an important role in many cases. It is relevant that metabolites from toxins generally possess ET functionalities which can participate in redox cycling. Much evidence exists in support of the theoretical framework. Toxic effects at the molecular level include lipid peroxidation, DNA attack, adduction, enzyme inhibition, oxidative attack on the CNS, and cell signaling. The toxins fall into many categories, including drugs, industrial chemicals, abused drugs, reproductive toxins, metal compounds, pesticides, and herbicides. Beneficial effects of AOs are documented, which may prove clinically useful. Knowledge of mechanisms operating in CNS insults should prove useful in drug design. A related update was reported in 2012 [6]. A similar article deals with nitric oxide (NO), catecholamine and glutamate [7]. The review treats the mechanism of these agents as important neurotransmitters and as neurotoxins, based on involvement of ET-ROS-OS. The unifying framework can be applied to both neurotransmission and neurotoxicity. Cell signaling, electrochemistry, AOs and apoptosis are also discussed. ET functionalities are known to play an important role in bioaction by way of ET-ROS-OS [7]. The principal actors are quinone (Fig. **1**), metal ions (Fig. **2**), nitroso aromatic from ArNO_2_ (Fig. **3**), and conjugated iminium (Fig. **4**). ET frequently results in formation of superoxide (Fig. **5**) which acts as precursor of other ROS (Fig. **6**). 

It should be emphasized that physiological action is often multifaceted. Therefore, other factors are likely participating in the CNS realm. References are often representative. Original reports are sometimes found in the reviews or articles.

## EPILEPSY 

This condition is one of the oldest illnesses that afflict mankind. Extensive literature supports involvement of ROS-OS as documented in the following reports. Direct injection of iron salts into the brain can produce epilepsy-like convulsions and death of neurons accompanied by lipid peroxidation and generation of hydroxyl radicals [8]. A report deals with glutamate transporters and how they are related to epilepsy and OS [9]. Patients suffering from the condition show decreases in activity of SOD and GSH peroxidase (glutathione tripeptide with a thiol group) [10]. Treatment with α-tocopherol showed reduced frequency of seizures and increased SOD activity, thus enhancing the therapeutic effect. Changes in AO defense mechanisms and resulting increased lipid peroxidation are involved in the pathogenesis [11]. Results indicate that the oxidant-AO balance is modified by antiepileptic therapy. Another report deals with the effect of SOD on seizures in an experimental model [12]. Only recently has it become known that OS and ROS generation are the cause and consequence of epileptic seizures [13]. Selenium and GSH peroxidase appear to play important roles in the pathogenesis in this context. Decreasing the TBARS (thiobarbituric acid reactive substances) elevation and increasing the SOD attenuation may be important characteristics of antiepileptic agents [14]. These effects may operate through an AO mechanism. Results indicate an oxidant-AO disturbance in epileptic patients, which can play an important role [15]. The patients showed reduced activity of various AOs and elevated lipid peroxidation. OS in all brain regions was associated with experimental epilepsy [16]. GSH plays a major AO role in certain regions. Certain semicarbazides were found to combat OS in epilepsy making them promising agents for therapy [17]. Cystatin B gene is mutated in one of the most common forms of epilepsy [18]. Its deficiency may couple OS to neuronal death and degeneration, thus providing the basis for novel treatment. The concentration of TBARS was increased and total AO status was decreased in epilepsy, supporting a role for OS [19]. Treatments designed to prevent lipid peroxidation may be more effective for epilepsy prophylaxis than administration of antiepileptic drugs that mask convulsive seizures while brain injury continues [20]. Melatonin exhibits AO, antiexcitotoxic and radical scavenging abilities [21]. Data indicate that the compound exerts AO activity in epileptic patients. Results suggest that decreased lipid peroxidation plays a role in the antiepileptic effect of oxcarbazepine [22]. Free radical production has been linked to epileptiform activity and seizure-induced cell death [23]. AO free radical scavengers reduce seizure-induced neuronal death. An investigation revealed AO properties of an extract from *Zizyphus jujube* in ameliorating seizures, OS and cognitive impairment in rat experimental models of epilepsy [24]. Increase in iron, a producer of ROS in epileptic patients, leads to an increase in OS [25]. The relationship between free radicals and scavenging enzymes has been established in epileptic phenomena, and ROS have been implicated in seizure-induced neurodegeneration [26]. There is evidence of lipid peroxidation and nitrite formation during seizure activity that could be responsible for neuronal damage during epilepsy. Increased lipid peroxidation may be causally related to structural abnormality in epilepsy [27]. Most of the epileptic women in an investigation had excess OS as indicated by high levels of MDA (malondialdehyde) [28]. Mitochondrial OS and dysfunction are contributing factors to various neurological disorders, including epilepsy [29-32]. Oxidative damage to cellular targets may affect neuronal excitability. A review examines the evidence for a role for oxidative injury in epilepsy, involving a rationale for AO therapy, and provides an appraisal of the current clinical status of AO therapy [33]. The low AO status in epileptic patients provides evidence for free radical involvement [34]. Lipid peroxidation was increased in kindled rats along with cell death [35]. AOs prevented the rise in lipid peroxidation. Results indicate involvement of ROS generated by NADPH oxidase in neuronal death in an epilepsy model [36]. A report suggests that OS arises in an epilepsy model from an increase in oxidant species, rather than from depletion of AO defenses [37]. Evidence exists for a role of Se and deficiency of GSH enzyme activity in epilepsy pathogenesis [38]. The role of SOD in a kindling model of epilepsy was investigated [39]. Seizure susceptibility and brain sensitivity to OS were studied in a comparison of male and female rats [40]. A study indicates the anticonvulsant and AO effects of curcumin and Nigella saliva oil in reducing OS, excitability and induction of seizures [41]. The role of lipid peroxidation was examined in the possible involvement of monamine oxidase in the epileptic rat brain [42].

## DOWN’S SYNDROME (DS)

One medically important area in which an imbalance in AO defense systems may be important is Down’s syndrome (DS) [8b], OS is suggested to be involved. An imbalance between CuZn SOD activity and H_2_O_2_-removing enzymes may play a role. There are considerably more recent relevant reports. 

Down’s syndrome is a congenital birth defect responsible for mental retardation [43]. An altered AO ratio may contribute to the condition. Aerobic exercise reduces OS in patients [44]. Data suggest that OS appears to be the consequence of low levels of reducing agents and enzymes that remove hydrogen peroxide [45]. Findings suggest importance in AO status which may be responsible for cataract formation in DS [46]. A pathological cascade leads to OS and neurodegeneration [47]. A misbalance between SOD and GSH peroxidase activity has been linked to free radical generation. The development of dementia in DS patients is paralleled with decrease in SOD activity [48]. A report points to increased OS and reduced cytokine signaling in DS [49]. Although there is evidence for OS, a conclusion states that AO supplementation is ineffective as a treatment for dementia in DS patients [50]. The OS which is present appears early in the fetus [51]. DS brain tissue is considered to be susceptible to oxidative injury, mainly because the increased SOD activity is not followed by an adaptive rise in enzymes that metabolize hydrogen peroxide [52]. The condition is characterized by increased lipid peroxidation, oxidative damage to DNA, and a rise in 8-OHdG, protein carbonyl and 3-nitrostyrene [53]. An elevated production of hydrogen peroxide mediates the increased rate of apoptosis in cells from DS individuals [54]. Evidence points to increased mitochondrial SOD in DS leading to elevated ROS [55]. There is a systemic decrease of all GSH forms in the blood of children with DS [56], who had severe reduction of vitamin C, slight reduction of vitamin E and slight rise in TBARS [57]. Abnormal maternal folic acid-homocysteine relates to oxidant/AO imbalance. Increased neuronal cell death in neurodegenerative diseases, such as DS, has been attributed to increase in ROS from mitochondria [58]. Results from a mice model suggest an increase in basal generation of ROS in neurons, probably caused by deficiency in mitochondrial ET. Oxidative imbalance in DS individuals may contribute to accelerated aging [59, 60]. Oxidative damage to protein confirms the role of OS in DS [61]. A 2010 review provides evidence for involvement of OS in DS and other genetic disorders [62]. As a result, research on chemoprevention with mitochondria-targeted supplements is warranted. Neuronal OS was found to precede amyloid-beta disposition in DS [63]. The AO system in DS is implicated in the cognitive phenotype associated with the disorder [64]. Increase in SOD, TBARS and catalase in people with DS supports the use of AO therapy [65]. Increased OS in DS was indicated by rise in protein oxidation and lipid peroxidation with reduction in GSH [66]. The oxidative damage is an early event. A report states that no scientifically proven drug or diet is yet available [67]. Peroxiredoxins are AO enzymes involved in protection against oxidative injury [68]. Under expression of the enzyme is observed in DS patients, which may contribute to the altered redox state. The pathogenesis apparently involves ROS which elevates SOD activity [69]. The patients have to cope with a significant prooxidant environment. OS causes alterations, such as atherosclerosis, early aging, neurological disorders, and immunological default. Increased hydroxyl radical formation in DS-gingival fibroblast was decreased by scavengers of hydrogen peroxide [70]. This may be due to the enzymatic ability of over-expressed SOD in DS to catalyze the formation of hydrogen peroxide from superoxide. OS affecting thyroxin biosynthesis may explain the proneness of DS patients to develop hyperthyroidism [71]. Selenium-containing proteins may be involved in thyroid hormonal synthesis by protection against ROS toxicity. The free radical dependent oxidation of uric acid to allatoin is apparently potentiated by OS in DS [72]. A recent review substantiates the claim that total AO capacity is decreased in adults with DS [73].

## ATAXIA

Ataxia involves impaired movement due to loss of motor coordination [8c]. Friedreich’s ataxia is the most common hereditary type. Early research resulted in pro and anti effects by vitamin E. Numerous reports show beneficial effects by countering ROS-OS. There is elevated cancer risk. The most severe manifestation is cardiomyopathy [74]. A cellular model shows that GSH peroxidase mimetics may provide a novel treatment strategy. Increased generation of ROS and mitochondrial dysfunction may underlie the psychophysiology of ataxia [75]. Impairment *in vivo* of AO enzymes provides evidence of increased OS, supporting a role of free radical cytotoxicity in the disease. Results provide additional evidence of constant OS in ataxia patients [76], AO treatment impairs *in vivo *cardiac and skeletal muscle energetics in ataxia patients [77, 78]. Ataxia cells exist under a constant state of OS with high levels of ROS which are removed by cellular AOs [79]. The suggestion was made that AO vitamin intake may modify the effect of breast cancer risk among ataxia patients. Alpha-tocopherol transfer protein maintains the concentration of this AO [80]. Vitamin E supplementation suppressed lipid peroxidation and almost completely prevented the development of neurological symptoms. After consideration of various factors, a review concludes that OS does constitute a major contributing factor to ataxia pathology and that AOs will be beneficial [81]. A similar conclusion was reached in another report [82]. In a study of AO therapy, N-acetyl-2-cycteine reduced both the incidence and multiplicity of lymphomas [83]. Decreased AO capacity and increased susceptibility to OS contribute to neuronal cell death in ataxia [84]. Oxidative damage of various types has led to the use of AOs, such as idebenone for therapy [85]. Vitamin E and CoQ10 therapy has proved beneficial in some patients associated with deficiencies in these AOs [86]. Other AO strategies involve the use of idebenone, Se and N-acetyl cysteine. But only idebenone has involved structured trials with relatively large number of patients, resulting in a clear impact upon cardiac hypertherapy in most cases. Scavenging of hydrogen peroxide by AO rescues frataxin deficiency in an ataxia model [87]. There are related investigations [88-90]. The ameliorating effect of L-carnitine pretreatment on oxidative DNA in ataxia was more prominent than for mannitol [91]. Mitochondria-targeted AOs protect ataxia fibroblast more effectively than untargeted AOs [92]. Treatment with the AO idebenone decreased 8-OHdG concentration [93]. Impaired mitochondrial metabolism with increased iron levels associated with increased ROS and OS are possible mechanisms that compromise cell viability [94]. Detoxification of ROS could occur *via *activation of GSH peroxidase and elevation of thiols. Iron loading occurs in the mitochondria of ataxia patients and may play a role in the pathogenesis [95]. Iron chelation was as effective as or more effective than conventional radical scavenges at protecting against peroxide-mediated cytotoxicity. Results indicate that idebrenone and vitamin E may act synergistically to counteract OS in ataxia fibroblasts [96]. A new class of porphyrinic radioprotectants was identified for general ataxia populations, which may provide a new option for treatment [97]. Administration of Se supplements should normalize the AO activity of GSH peroxidase, thus slowing the progression of cardoiomyopathy associated with ataxia [98]. Treatment with a catalytic AO corrects the neurobehavioral defect in ataxia mice [99]. A report confirms that non-prescription AO use represents a major confounder to formal trials of therapeutic agents [100]. 

There is additional literature dealing with ROS and OS in ataxia. Friedreich ataxia, the most common hereditary form, is caused by a deficit in the mitochondrial protein frataxine [101]. Data support an important role for OS in the disease progression and suggest sensitivity to frataxine imbalance. Oxidative damages to Fe-S clusters, resulting from hampered SOD signaling, may play a role [102]. In Friedreich’s ataxia, loss of mitochondrial frataxin causes iron overload and increased ROS leading to oxidation and inactivation of the respiratory chain enzymes [103]. Findings suggest that iron metabolism is dysregulated in ataxia [104]. Iron chelators increase the resistance to OS. A recent article supports the view that mitochondrial dysfunction and OS contribute to the pathogenesis of ataxia [62,105]. 

## MULTIPLE SCLEROSIS (MS) 

A 2000 book reviews prior work in the OS area [8d]. The disease, believed to be autoimmune, is characterized by impaired nerve conduction due to demylination. There have been repeated reports that oxidative damage contributes to MS pathology. Activated macrophages will generate ROS and possibly NO. Nitrotyrosine has been reported in the MS brain. Several studies claim that AOs alleviate some of the damage in animal models, although results vary.

State of lipid peroxidation and activity of AO defense-enzymes were investigated with MS patients [106]. Development of OS is progressive involving intensive lipid peroxidation. These conditions are accompanied by high activity of demylination and severe neuronal injury. Development of OS is a result of strong metabolic disorders and decrease of AO defenses. Approaches to AO therapy are discussed. Efficiency of AO therapy was reported for relapsing MS patients [107]. Treatment with mixtures of AOs and neuroprotectors resulted in significant reduction of relapse frequency [107]. After AO therapy, the content of lipid peroxide products was reduced. Increasing data indicate that OS plays a major role in MS [108]. ROS, generated by microglia/macrophages, are implicated as mediators of demylination and neuronal damage. ROS cause insults to many cellular constituents, accompanied by weakened AO defenses. The CNS is particularly susceptible to ROS-induced adverse effects due to high oxygen demands in the brain and low concentrations of AOs. Hence, benefits may arise from use of exogenous AOs. Related studies have been reported dealing with AO protection [109-124]. Sensitivity of motor neurons to reactive nitrogen species has implications for MS [125]. Additional evidence suggests a central role for OS in MS [126]. MS patients display elevated TBARS levels, reduced protein SH groups and slightly reduced SOD [127]. Methylprednisone protects against free radical attack. Another study deals with OS involvement [128]. Neurodegenerative diseases, including MS, are initiated by microglia activation and mediated by OS and excitotoxicity [129]. OS leads to lipid and protein damage *via *peroxidation and nitration [129]. The inflammatory component in MS is important for the design of therapeutic strategies. There is increase in OS in patients with remitting MS [130]. NO metabolites, lipid peroxidation products and GSH peroxidase activity are significantly increased in subjects with this condition. A report deals with mechanism of OS toxicity involving sphingomyelinase in MS [131]. Nitrite levels in blood leukocytes of MS patients and measures of OS are clearly related [132]. Increased NO levels may be the result of reaction to OS. It is well known that excessive OS and neurotoxicity are involved in MS [128]. Electrical stimulation has been used in recovery. After 8 months of such treatment, the patient’s function had considerably improved. Reports also deal with potential therapy and early management of MS [133-135].

A 2011 review addresses inflammatory disorders and their redox control from molecular mechanisms to therapeutic opportunities, including neurodegenerative diseases, such as MS, ALS, Alzheimer’s and Parkinson’s [136]. Discussion involves the identity, source, regulation and bioactivity of redox molecules. Also, potential therapeutic implications involving novel drugs to combat chronic inflammatory disorders are treated. Studies of neuroinflammatory disease, e.g. MS, suggest that the intracellular level of GSH may play a critical role in the regulation of cytokine-induced generation of ceramide leading to apoptosis of brain cells [137]. Syncytin-mediated neuroinflammation and death of oligodendrocytes, with the ensuing neurobehavioral defects, were prevented by the AO ferulic acid in a mouse model of MS [138]. Thus, syncytin’s proinflammatory properties in the nervous system demonstrate a novel role for a retrovirus protein, which may be a target for therapeutic intervention. Within the brain, the susceptibility of brain cells to NO and peroxynitrite may be dependent on factors, such as GSH and stress-resistance signal pathways [139]. Hence, neurons appear particularly vulnerable to the effect of nitrosative stress. Evidence is available to support this scenario for neurological disorders, such as MS, ALS, Alzheimer’s, Parkinson’s, Huntington’s, Down’s syndrome and other types of brain damage. The AO bilirubin could represent a protective system potentially active against brain oxidative injury. Increasing evidence indicates that the heme oxygenase-1, and its expression appears closely related to conditions of oxidative, nitrosative stress and oxidative DNA damage [140,141]. The clinical manifestations of MS might be explained by an oscillating process of oxidative change of the quasi catalytic function of melanin pigments from destroying ROS to transforming them to more harmful, longer lasting species [142]. The suggested unifying concept of MS pathogenesis may open perspectives for prevention, diagnosis and therapy. Axonal damage and neuronal loss are significant pathological components of MS [143]. Altered redox homeostasis and increased OS may be primarily implicated in the pathogenesis. A study was made of the regulation of redox forms of plasma thiols in MS [144]. Plasma levels are subjected to complex redox changes by oxidation and thiol/disulfide exchange reactions.

## HUNTINGTON’S DISEASE (HD)

Earlier studies of this condition indicated involvement of ROS/RNS [8e]. The disease is inherited and characterized by psychiatric disorders, dementia, twitchings, writhings and death of neurons. Levels of 8-OH dG are known to increase in the HD brain. There is some evidence for a role of excitotoxicity. The excitotoxin quinolinic acid is normally present in the brain. The concentration of the enzyme involved in its synthesis is raised in HD.

The neurodegenerative disease mainly affects the basal ganglia [145]. OS is involved in the pathogenesis of HD, as is the case for various other neurodegenerative conditions. OS may be used as a marker of both the disease and therapeutic effectiveness. The origins of the prevalent OS have been investigated [146]. One source appears to be mitochondrial dysfunction leading to overproduction of ROS. There may be other sources. Decreased levels of a major intracellular AO GSH coincide with accumulation of ROS in HD neurons. These neurons were also deficient in the AO cysteine. Another report demonstrates a role for free radicals in OS involving HD [147]. Other findings support a role for free radicals in the onset and progression of the disease [148]. Changes in ROS formation may be due to increased propensity of the striatum to generate the radicals as a response to the pathological conditions. Another investigation addresses participation of mitochondrial dysfunction [149]. Mitochondria play a central role in both metabolism and OS. Increasing evidence demonstrates mitochondrial abnormalities in HD for both mice and humans. OS, mitochondrial dysfunction and protein metabolism impairment have been implicated in the pathogenesis of HD [150]. Results indicate that therapy directed to counter OS may be useful in the treatment. A primary finding is the decreased activity of catalase in HD patients, suggesting participation of OS [151]. OS is more pronounced in HD patients and HD gene carriers than in healthy subjects [152]. The patients have higher lipid peroxidation levels and reduced GSH concentrations. Data suggest that OS occurs before the onset of HD symptoms. Lipid peroxidation data indicate a high level of OS in HD patients many years after disease onset [153].

Various studies support a role for AOs in alleviating HD. Data suggest that oxidative damage is a prerequisite for striatal lesion formation in HD and that AO treatment may be a useful therapy [154]. Results revealed an increase in OS biomarkers and a reduction in AO systems in HD patients [155]. OS stress was induced by 3-nitropropionic acid in rats serving as a model of HD [156]. The adverse effects were prevented by melatonin administration. The protective mechanisms to the neural response likely involve AO processes.

## EMOTIONAL STRESS 

Psychological stress, becoming increasingly common, is known to predispose to various diseases, including cardiovascular, gastric ulcers and infection. It has long been speculated that ROS are involved [8f]. Early work showed that stress induced in rats leads to decrease in the AO GSH and increase in protein carbonyl, lipid peroxidation and 8-OHdG from oxidative attack.

There are more recent, pertinent findings. It has been ascertained that there is a common pathogenic link, namely excessive production of free radicals, in the mechanism responsible not only for infections, but also for other factors, such as emotional stress [157]. In all cases, the same metabolic changes occur in different ways involving the production of higher quantities of active oxygen forms, such as NO and other radicals. The body responds, in part, *via *protection by AO generation. Emotional stress is clinically well known as a developing and exacerbating factor in autoimmune diseases [158]. In these conditions, excessive OS is believed to play a role by enhancing inflammation, inducing apoptotic cell death and breaking down immunological tolerance. The excess OS resulted in decreased AO defenses and increased amounts of 8-OHdG. In a study of the effect of stress, TBARS levels significantly increased in old monkeys [159]. Stress led to activation of lipid peroxidation, which is deemed an important factor. AO responses were also investigated. A study was made involving the influence of physical activity on emotionally stressed rats [160]. Results highlight the potential benefit of physical activity to reduce oxidative damage induced by emotional stress, since the activity attenuated protein oxidation and mitochondrial alterations. A report dealt with superoxide as a regulator of the cardiovascular response to emotional stress [161]. Growing evidence indicates that ROS, particularly superoxide, are important intracellular messengers of many brain actions, including cardiovascular response to emotional stress. This review by Rahman *et al*., summarizes knowledge of redox-sensitive signaling mechanisms in the brain that regulate cardiovascular effects of stress. AO effects are addressed in relation to psychological stress-induced OS and neurotransmitter status [162]. The protective action of anthocyanins was studied. The administered AO was active in the brain, suppressing stress-induced cerebral OS in distressed mice. The results suggest possible therapeutic use. Psycho-emotional stress in rats was accompanied by intensification of lipid peroxidation and reduced activity of AO enzymes, such as catalase and SOD [163]. The condition, involving OS, causes a reduction in the intensity of energy metabolism in cardiomyocytes. The results indicate that stress is one of the factors involved in cardiac diseases. Degenerative diseases, immune impairment and premature aging commonly affect certain professional categories of people exposed to severe environmental or psychological stress [164]. The free radicals/AO imbalance is a common feature of the pathological conditions. Alterations observed include OS, decreased AO levels, DNA oxidation and cardiovascular malfunction. Use of AO supplementation and diet regimen were discussed. Various studies established a link between OS and pathological anxiety [165]. A causal relation may exist between OS and emotional stress. The article by Goncharova *et al*., examines the link between OS and normal anxiety levels and the role of OS in genesis of anxiety. Methodological approaches are discussed that are being used to determine a causal relationship between OS and emotional stress. An investigation was made of age-related changes in reaction of erythrocyte AO enzyme systems in response to acute psycho-emotional stress, and a possible role of these changes in age-related alterations [166]. These alterations in SOD and GSH reductase responsiveness led to activation of peroxide oxidation of lipids that may be a factor in aging damage under stress conditions.

## AMYOTROPHIC LATERAL SCLEROSIS (ALS)

The earlier, relevant work has been summarized [8g]. ALS is a fatal neurodegenerative disease that mainly affects motor neurons in cortex, brainstem, and spinal cord. The majority of ALS cases are sporadic. However, in 5-10% of ALS cases, the disease in inherited as an autosomal dominant trait. Additionally, 15-20% of ALS are associated with mutations of the superoxide dismutase 1 gene, the main scavenger enzyme of the superoxide radical. There is considerable evidence that oxidative damage occurs in ALS. Protein carbonyl and nitrotyrosine are elevated in the spinal chord. NO could originate in sites of neural injury. Increase in the amount of iron and copper, catalysts for ROS production and OS lead to several intracellular alterations and contribute to the induction of cell death pathways [167]. Evidence suggests that mitochondrial superoxide dismutase activity and OS may be involved in the pathogenesis of ALS [168,169]. A study showed synthetic superoxide dismutase/catalase mimetics reduce OS and prolong survival in mouse models with ALS [170]. 

Oral administration of Neu2000, a potent antioxidant, blocked the increase in ROS and reduced the activation of proapoptotic proteins [171]. Administration of lithium carbonate, a mood stabilizer that prevents apoptosis, blocked the apoptosis machinery without preventing OS. Administration of Neu2000 and lithium produces marked improvement in motor neuron survival.

## PRION DISEASES (PD)

Prion diseases of transmissible spongiform encephalo-pathies are a group of neurodegenerative disorders including scrapie in sheep, bovine spongiform encephalopathy in cattle, Creutzfeldt-Jakob disease (see below), and Gerstmann-Sträussler-Scheinker disease in humans. In prion diseases, the normal cellular form of the soluble prion protein, which consists of α-helix and random coil structures undergo a conformational conversion to the ß-pleated sheet–rich scrapie isoform that is partially resistant to protease digestion [172]. The accumulation of prion protein, which occurs in the cytoplasm and in secondary lysosomes as well as in neuronal plasmalemma and synaptic regions, may be responsible for the loss of cognitive function in prion diseases. In the brains of animals at the terminal stage of illness, there is a marked decrease of prion protein, supporting the hypothesis that loss of function of prion protein may play a role in the pathogenesis [173,174]. The data indicate that the ß-cleavage of prion protein is an early and critical event in the mechanism by which prion protein protects cells against OS. Prion protein plays an important role in anti-oxidative defense and its deficiency increases susceptibility to OS [175,176]. A study showed that neurotoxic prion peptides stimulate N-methyl-D-aspartate accompanied by the release of arachidonic acid in cerebellar granule neurons, suggesting the association of phospholipase A2 in the pathogenesis of prion disease [172].

## TARDIVE DYSKINESIA (TD)

Prior to 2000 there have been only meager amounts of data relevant to the OS approach [8c]. TD is a serious neurological syndrome associated with long-term administration of neuroleptics to humans and experimental animals. This extrapyramidal disorder is characterized by repetitive involuntary movements, involving mouth, face, and tongue, and sometimes limb and trunk musculature. This syndrome is most frequently found in older patients using antipsychotic agents. OS and products of lipid peroxidation are implicated in the pathophysiology of this neurological disorder. A review deals with oxidative mechanism and TD [177]. Other reports also implicate OS related genes to TD [178-180]. AOs vitamin E [181], resveratrol [182], alpha lipoic acid [183], and quercetin [184] administration is beneficial to patients.

## ALZHEIMER’S DISEASE (AD)

A 2011 review deals with oxidative stress in neuro-degeneration, highlighting the role of OS in neurodegenerative diseases, including Alzheimer’s, Parkinson’s, Huntington’s and amyotrophic lateral sclerosis [185]. A similar report discusses free radicals and antioxidants in normal physiological functions and human disease, such as Alzheimer’s and Parkinson’s [186,187]. Neurodegeneration results from abnormalities in cerebral metabolism and energy balance within neurons, astrocytes, microglia, or microvascular endothelial cells of the blood-brain barrier. AD is characterized clinically by a progressive loss of memory and cognitive functions. Neuropathologically, AD is defined by the accumulation of extracellular amyloid-protein deposited senile plaques and intracellular neurofibrillary tangles made of abnormal and hyperphosphorylated tau protein, regionalized neuronal death, and loss of synaptic connections within selective brain regions [188]. Data showed aggregates of hyperphosphorylated tau protein binds to Fe3+, which induces neurofibrillary tangle production [189]. Redox-active iron (Fe^3+^/Fe^2+^ complexes) is deposited near the beta-protein plaques and neurofibrillary tangles in the cortex of AD brains. Study with transgenic mice showed mRNA oxidation may be associated with neuronal deterioration during the process of neurodegeneration [187]. More evidence suggests a critical role for amyloid-ß peptide metabolism and OS in AD pathogenesis and progression [190-193]. Among the other indices of OS in AD brain, protein carbonyls and 3-nitrotyrosine have been detected and used as markers of protein oxidation [188]. A report focuses on the effect of methylglyoxal, a cytotoxic byproduct of glucose metabolism and its interaction with amino acid residues within ß-amyloid, and small peptides within brain, leading to neurodegeneration [194]. A review discusses the application of redox proteomics in the identification of oxidatively modified proteins in AD pathology, suggesting redox potential targets for therapy [188]. 

In addition to extracellular neuritic plaques and intracellular neurofibillary tangles, OS and impaired mitochondrial function always accompany Alzheimer’s disease. Mitochondria are described as “cellular power plants” as they are responsible for the production of ATP and oxidative phosphorylation, signal transduction, control cell cycle, cell growth and programmed cell death. Dysfunction of mitochondrial energy metabolism leads to reduced ATP production, impaired calcium buffering, and generation of ROS, such as superoxide anions, hydroxyl radicals and hydrogen peroxide. Mitochondrial dysfunction may be the principal underlying event in aging, and age-associated brain degeneration. Evidence shows ROS induced mitochondrial damage and vascular hyperfusion as key initiators for the development of AD [195-197]. A report showed oxidation of potassium channels by ROS playing a role in neuro-degeneration [198]. Nanoparticles and colloids, which are mineral or proteinaceous materials of intrinsic or exogenous origin, may also contribute to the neurodegeneration [199]. AD is a progressive neurodegenerative disorder involving misfolding and aggregation of proteins in conjunction with prolonged cellular stress. Evidence shows that endoplasmic stress may play a role in the pathological changes [200]. Copper has been shown to have an important role in neurodegenerative diseases, such as Alzheimer’s, Parkinson and amyotrophic lateral sclerosis [201]. OS in the cardiovascular system, including brain microvessels and /or parenchymal cells results in an accumulation of ROS and RNS, thus promoting leukocyte adhesion and increasing endothelial cellular hypometabolism [202]. Authors suggest that hypometabolism, coupled with oxidative stressors, is responsible for AD and cerebrovascular accidents. 

Over the years a number of reports have appeared on AO based therapy and protection against OS and neuro-degenerative diseases, including AD [185, 201-210].

## PARKINSON’S DISEASE (PD)

PD is a common neurodegenerative movement disorder which is clinically characterized by progressive rigidity, bradykinesis, and tremor. Pathlogically, PD is characterized by loss of melanin-pigmented nigral neurons, accompanied by depletion of dopamine in the striatum and the presence of Lewy bodies [185]. As in the case of other neuro-degenerative disorders, mitochondrial dysfunction, oxidative damage, environmental factors, and genetic predisposition may all be involved in both sporadic and familial PD [185, 211-213]. Postmortem tissues from PD patients have shown evidence of a defect in complex I of the mitochondrial electron-transport chain in substantia nigra, resulting in a 30-40% decrease in the activity, which may be the central cause of sporadic PD [185]. Evidence also showed oxidative damage to DNA, protein nitration, glycation, and the lipid oxidation product 4-hydroxy-2-nonenal in PD brains [185] Ceruloplasmin is an extracellular ferroxidase that regulates cellular iron loading and export, and protects tissues from oxidative damage. A report showed ceruloplasmin oxidation leading to decrease in ferroxidase activity, which in turn promotes intracellular iron retention in neuronal cell lines, as well as in primary neurons in PD [214]. A recent review deals with metal attenuating therapies on neurodegenerative disease [215].

## CREUTZFELDT-JACOB DISEASE 

This condition (CJD) falls into the prior disease categories which has been associated with OS [8h]. Findings provide evidence for a link between aberrant expression of AO proteins and CJD pathogenesis [216]. The neuropathological measuring of AO proteins in CJD brain is discussed. Fragmentation of the neuronal Golgi apparatus was reported in CJD [217]. The mechanisms involved implicate dysregulation and disruption by mutant SOD [218].

## ROLE OF IRON

A recent review presents a unifying theme for cellular death and neurotoxicity by iron agents [219]. Among the many neurodegenerations are Parkinson’s, Huntington’s, Alzheimer’s, prions and others. The basic theme involves continuing and autocatalytic generation of hydroxyl radicals by way of the Fenton reaction involving poorly liganded iron. The process is multifaceted, also entailing cell signaling. Multiple protectors, such as iron chelators and AOs, are also likely to prove effective.

The effects of iron in various diseases are as follows:

### ALS

Iron has been strongly implicated in this neurodegenerative disease. The positive effects of iron chelators in mouse models and others suggest initiation of human trials. There is a connection between some ALS and mutation in genes coding for SOD.

### Huntington’s Disease (HD)

HD proteins contain inclusion bodies that act as centers of OS resulting in large amounts of oxidase proteins. Iron chelation proved protective.

### Parkinson’s Disease

There is substantial evidence for iron involvement. Iron chelators inhibit these inimical reactions. Proteins are oxidatively modified. The neuromelanin present binds large amounts of iron that appears to act as a Fenton catalyst. Lipofuscin, a dark pigment commonly present, also contains oxidized proteins and lipids, and can absorb high amounts of iron.

### Alzheimer’s Disease

AD, the commonest one in this category, shares many similarities with the others, including a role for OS. Generation of insoluble polymers, such as, β-amyloid, can lead to complexation with iron, followed by ROS-OS. Iron chelators are known to ameliorate fibril formation, neurodegeneration, dementia and AD. Human trials based on these observations should be worthwhile. 

### Ataxia

Fenton chemistry, based on bound iron, is strongly implicated. Iron chelation may lead to therapeutic benefits. Attenuation of hydrogen peroxide production ameliorates the adverse condition.

### Prion Disease

Prion protein binds iron tightly, resulting in conversion to a form that can sequester the metal in insoluble ferritin complexes. Iron metabolism is modified in the disease. Hydroxyl radical formation has not been reported. There is evidence for iron involvement, including metabolic dysregulation in the OS-mediated etiology of scrapie. Geostatistics relate variation of metals present in soils to prion disease prevalence. 

### Other

Additional information on iron as a toxic, redox metal is available in a recent review [219]. 

## ABBREVIATIONS

ET: = Electron transfer
ROS: = Reactive oxygen species
AO: = Antioxidants
OS: = Oxidative stress
DS: = Down’s syndrome
MS: = Multiple sclerosis
HD: = Huntington’s disease
ALS: = Amyotrophic lateral sclerosis
PD: = Prion disease
TD: = Tardive dyskinesia
AD: = Alzheimer’s Disease
PD: = Parkinson’s Disease
TBARS: = Thiobarbituric acid reactive substances
GSH: = Glutathione
MDA: = Malondialdehyde
